# Recurrent Ischemic Strokes due to Monogenic COL4A1 Mutation: The First Case Report from Latin America

**DOI:** 10.1155/2023/6614837

**Published:** 2023-08-30

**Authors:** Emilio Israel Wong-Valenzuela, Daniel San Juan, José Santos Zambrano, Alejandra Camacho Molina, Miguel Angel Morales-Morales, Alejandro Lopez-Landa

**Affiliations:** ^1^Epilepsy Clinic, Instituto Nacional de Neurologia y Neurocirugia, Mexico City, Mexico; ^2^Neurology Department, Hospital Medica Sur, Mexico City, Mexico; ^3^Rare Diseases National Program, Institute for Social Security and Services for State Workers, Mexico City, Mexico; ^4^Faculty of Medicine, Benemérita Universidad Autónoma de Puebla, Puebla, Mexico

## Abstract

*Introduction*. Monogenic mutations as the cause of recurrent ischemic cerebral small-vessel disease with leukodystrophy are rare. COL4A1 gene mutations are a relatively new etiology of cerebrovascular lesions in young adults; however, any patient has been reported from Latin America. *Case Presentation*. We presented a Mexican young female with leukodystrophy and recurrent stroke secondary to COL4A1 monogenic mutation. *Discussion/Conclusion*. COL4A1 monogenic mutations are associated with cerebral small-vessel disease and other systemic manifestations. To date, there is little evidence to justify the treatment and prevention of recurrent strokes in patients with this mutation.

## 1. Introduction

Cerebral small-vessel disease (SVD) is one of the main causes of cerebral infarction and cognitive deficit to date. Most cases are sudden, and polygenic variations have been shown to be associated with this disease; however, cerebral SVD is not a common cause of infarction in young adults, and recently, more monogenic entities are the cause of SVD in this population. The most common and best known of these mutations is in NOTCH3, which results in cerebral autosomal-dominant arteriopathy with subcortical infarcts and leukoencephalopathy (CADASIL) [1].

In 2005, mutations in the type IV collagen coding gene, in either COL4A1 or COL4A2, which are responsible for encoding the pro*α*1(IV) and pro*α*2(IV) chains, respectively, were observed to be related as a cause of porencephaly in mice [2], and since then, mutations in these genes were initially associated with autosomal-dominant porencephaly and infantile hemiparesis and currently recognized as a condition with neurological manifestations such as ischemic and hemorrhagic stroke, migraine and seizures, and systemic features such as ocular, renal, and muscular involvement; nevertheless, recurrent ischemic stroke is one of the most common phenotypes in young adults debuting with cerebral SVD [1, 3] and any patient has been previously documented from Latin America [4].

Here, we present the first Mexican young female with leukodystrophy and a history of recurrent cerebral SVD secondary to a COL4A1 monogenic mutation.

## 2. Case Presentation

A 35-year-oldright-handed female, who was born as the second daughter of a 26-year-old mother who had preeclampsia and was born by C-section, prematurely at 7 months. She breathed and cried at birth, remained hospitalized in an incubator for 2 weeks, and was discharged without any complications. She had a normal development and had an obstetric history of two pregnancies complicated by severe preeclampsia, which culminated in the early termination of pregnancy. Furthermore, she has family history of unspecified heart disease and systemic arterial hypertension from her maternal grandmother and her mother with a history of preeclampsia with left cerebral infarction and chronic right hemiplegia related to the birth of the patient.

Four years prior to our initial evaluation, she had the first ischemic stroke triggered by emotional stress while she was going to sleep. The next day, she noticed slurred speech and mild right hemibody weakness. She continued her normal activities for two days until the condition worsened and she was unable to write, walk, or perform her normal activities. Her physical examination showed right facial hemibody hypoesthesia, with strength of 4/5 in the right arm and 3/5 in the right leg and hyperreflexia in the right hemibody. The initial workup approach showed an increase in creatine phosphokinase (595 U/L), and complete blood cell count and liver, renal, and serum electrolyte tests were normal. The brain 1.5TMR scan showed a posterior pattern of leukodystrophy and acute lacunar infarction in the body of the left caudal nucleus. Carotid and renal Doppler ultrasonography was performed, where a decreased mild flow of the left carotid artery was reported, with normal kidney circulation. Electrocardiogram and electromyography with nerve conduction studies with late F and H responses were normal. She was hospitalized for 1 week and received empirical treatment with intravenous methylprednisolone boluses (1 g per day for 3 days) with partial neurological improvement and a genetic complete exome sequencing (CentoXome GOLD) test was ordered, and a change in direction mutation in COL4A1 (variant coordinates; Chr13 [GRCh37]: g.110828842C>T; NM_001845.4:c.2987G>A; p.(Gly996Asp) and Exon 36) was found **(**Table 1**)**. No other clinically relevant genetic variant was reported according to ACMG guidelines. After one year of receiving 100 mg acetylsalicylic acid, physiotherapy, and speech therapy, she recovered 80% of her lost strength and 100% of her speech.

**Figure 1:**
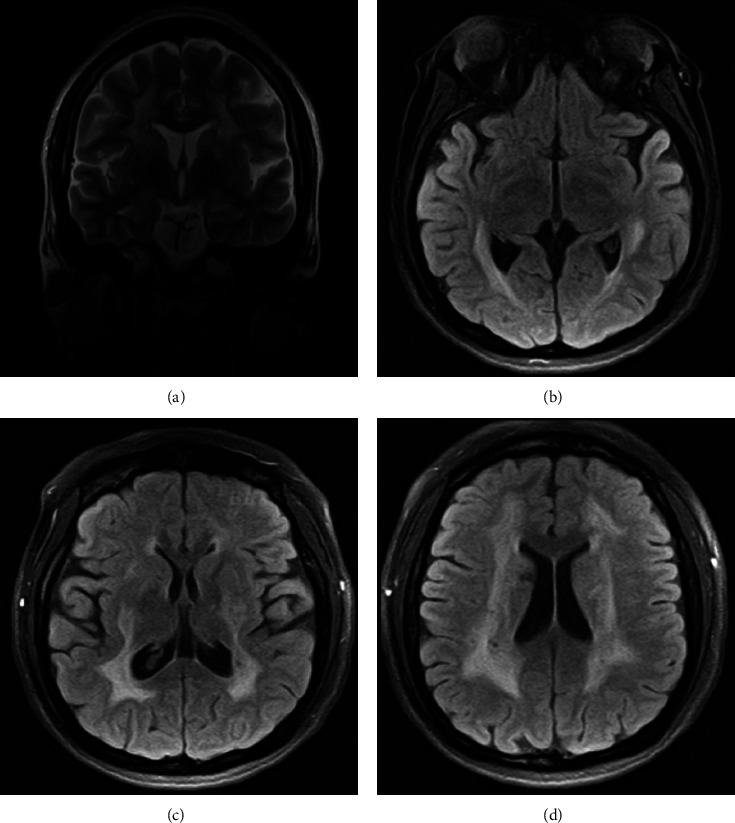
Brain 3.0 Tesla MRI. (a) Coronal section in the T2 sequence. (b–d) Axial sections in T2 FLAIR sequence showing a bilateral and symmetric posterior leukodystrophy, acute right frontal and parietal lacunar ischemic strokes, and chronic lacunar infarction in the left body of the caudate nucleus.

Three years after her initial stroke, she suffered from an upper respiratory tract infection and received erythromycin (500 mg every 8 hours for 7 days). During this treatment, she had an episode characterized by loss of balance and worsening of the right hemiparesis. Her brain MRI **(**Figure 1**)**, FLAIR, and T2 sequences showed posterior leukodystrophy, acute right frontal and parietal lacunar ischemic strokes, and chronic lacunar infarction in the left body of the caudate nucleus and she was hospitalized again to receive IV methylprednisolone in the bolus with recovery to the baseline. She was discharged with 75 mg oral clopidogrel and 60 mg oral pravastatin for 6 months.

## 3. Discussion

We presented the first Latin American young female with leukodystrophy and recurrent cerebral SVD secondary to COL4A1 monogenic mutation.

Monogenic Mendelian disorders are the cause of stroke in young patients for up 7%, and a large proportion of these ischemic strokes are recurrent and confluent cerebral small-vessel disease affecting mainly white matter without any other common vascular risk factor [5]. More than 35 monogenic variations can give rise to SVD and leukoencephalopathy with autosomal-dominant, autosomal-recessive, or X-linked inherence patterns. Currently, CADASIL is the most common hereditary SVD [1]. However, a recent review analyzed 390 individuals with heterozygous mutations in COL4A1 and stroke (41%) was the most frequent phenotype, followed by other neurological manifestations as cerebral aneurysm (36%), cognitive features (33%), seizures (32%), or intracerebral hemorrhage (ICH) (7%) [4]. Cases of familial recurrent ICH have also been reported [3].

There are other clinical entities related to mutations in the COL4A1 including porencephaly that may or may not be associated with visual disturbances, as well as systemic manifestations such as kidney damage, Raynaud's phenomenon, cardiac arrhythmias, muscular abnormalities, and hemolytic anemia [6]. There is a slight predominance for female with a median age at the time of assessment of 17 (range ≤1–77) years [4]. However, COL4A1 and COL4A2 mutations have a wide intra- and interfamilial variations with reduced penetrance; however, de novo mutations can be found in up to 42% of patients, which present with more severe forms of the disease [3]. In our patient, we only found an elevated CPK levels as systemic manifestation; however, a muscle biopsy was not performed because the electromyography was normal and other studies were not relevant for the management.

The type IV collagens are encoded mainly by two genes: collagen type IV alpha 1 (COL4A1) and collagen type IV alpha 2 (COL4A2). COL4A1 is located on chromosome 13q34 coding for type IV collagen, and this type composes the basal membranes in blood vessels and organs such as eyes, muscles, kidneys, and brain. A mutation in an untranslated region of COL4A1 causes an upregulated overexpression of this gene, resulting in the intracellular accumulation of collagen and the extracellular deposition of defective collagen causing small cerebral vessels to be fragile [4]. Microscopy showed the proliferation of intima with increased number of elastic fibers and atrophy of the tunica media of small arteries and arterioles, evidenced in the patients as lacunar infarcts due to frugality of the vessel walls [7].

It should be mentioned that mutations in this gene are autosomal dominant [8], and in the case of our patient, a class 2 variation was identified. There is an unclear history of probable cerebral stroke in her mother; however, genetic studies have been offered to her relatives to confirm and results are yet to come.

The neuroimaging markers of CT or MRI are emblematic in the suspicion of a cerebrovascular event in young patients, and the presence of lesions characteristic of SVD such as cerebral atrophy, leukoaraiosis, white matter hyperintensities, and lacunar microinfarcts allows visualizing a genetic pathophysiological picture [9]. Symmetric white matter involvement is a fundamental finding of leukodystrophy, which suggests the association with a hereditary disorder, the main markers being hyperintensity on T2-weighted and hypointense T1-weighted images, although the patterns of presentation are variable [10]. The most common brain MRI findings in COL4A1 mutations are periventricular leukoencephalopathy, porencephaly, cerebral calcification, or microbleeds [3]. The differential diagnosis for this leukoencephalopathy findings includes autoimmune disorders such as lupus erythematous systemic and other primary and secondary vasculitis; however, the diagnosis in our case is confirmed by the mutation found in the complete exome sequencing in COL4A4, which consists of 52 exons, and the most frequent findings are missense mutations involving highly conserved glycine residues in the triple helicoidal domain of this gene [6]. Genetic diagnosis can also be based on targeted gene panels [11].

Regarding the management of patients with this disease, it is recommended to avoid sports that carry a high risk of traumatic brain injury and inflammation appears to be a key mechanism of recurrence, which makes treatment with corticosteroids a promising approach [12]; however, most of the recommendations available are based on CADASIL, so it requires clear and updated recommendations for patients with monogenic mutations in COL4A1.

## Figures and Tables

**Table 1:** Summary of CentoXome GOLD results.

Gene	Variant coordinates	Zygosity	*In silico*parameters^*∗*^	Allele frequencies^*∗∗*^	Type and classification^*∗∗∗*^
COL4A1	Chr13(GRCh37):g.110828842C>T	Het	PolyPhen: probably deleterious	gnomAD: -	Change in direction
	NM_001845.4:c.2987G>A		Align-GVGD: C65	ESP: -	Probably pathogenic
	p.(Gly996Asp)		SIFT: Deleterious	1000 G: -	
	Exon 36		MutationTaster: Pathogenic	CentoMD: -	Class 2
			Conservation: nt high/aa high		

Variant description based on the Alamut batch (latest version available). ^*∗*^AlignGVD: C0: lower probability of interfering with function, C65: higher probability of interfering with function; splicing predictors: SSF, MaxEnt, HSF. ^*∗∗*^genome aggregation database (gnomAD), exome sequencing project (ESP), 1000genome project (1000G), and CentoMD (latest version available). ^*∗∗∗*^based on ACMG recommendations.

## Data Availability

The data generated or analyzed during this study are included within this article. Further enquiries can be directed to the corresponding author.
